# Factors for late initiation of antenatal care in Dar es Salaam, Tanzania: A qualitative study

**DOI:** 10.1186/s12884-019-2576-0

**Published:** 2019-11-12

**Authors:** Saidi Mgata, Stephen Oswald Maluka

**Affiliations:** 1 Malaria Programme Laboratory, Walter Reed Program-Tanzania, P.O.BOX 13303, Dar es Salaam, Tanzania; 2 Institute of Development Studies, University of Dar es Salaam, P.O.BOX 35169, Dar es Salaam, Tanzania

**Keywords:** Antenatal care, Late attendance, Tanzania

## Abstract

**Background:**

Antenatal care (ANC) provided by a trained health care provider is important for monitoring pregnancy thereby reducing potential risks for the mother and child during pregnancy and delivery. The World Health Organization (WHO) recommends at least four ANC visits to all pregnant women. While the proportion of women who attend at least one ANC in low-income countries is high, most pregnant women start their first ANC attendance very late. In Tanzania only 24% of pregnant women start their first ANC attendance before the fourth month of pregnancy. While factors for the utilization of antenatal care in general have been widely studied, there is paucity of studies on the factors affecting timing of the first ANC attendance. This study aimed to understand individual, community, and health system factors that lead to the delay in seeking ANC services among pregnant women in Ilala Municipal in Dar es Salaam region, Tanzania.

**Methods:**

A qualitative exploratory study, using in-depth interviews with 20 pregnant women and five health care workers was conducted in three different health facilities in Dar es Salaam Tanzania. Thematic analysis approach was used to analyse the data.

**Results:**

Individual perceptions of antenatal care, past experience with pregnancy, fear of pregnancy disclosure, and socio-cultural beliefs were the key individual and social factors for late ANC attendance. Shortage of trained health care workers, lack of spouse’s escort and health providers’ disrespect to pregnant women were the main health system barriers to early ANC attendance.

**Conclusions:**

This study concludes that community members should be sensitized about the importance of early ANC attendance. Additionally, while spouse’s escort policy is important for promoting PMTCT, the interpretation of the policy should not solely be left to the health providers. District and regional health officials should provide correct interpretation of this policy.

## Background

Antenatal care (ANC) provided by a trained health care provider is important for monitoring pregnancy, thereby reducing potential risks for the mother and child during pregnancy and delivery. Approximately 300,000 women die each year due to pregnancy related deaths and over 99% of these deaths take place in low income countries and most could have been prevented [1].While the timing of ANC varies between high and low income countries, there is almost common understanding that ANC attendance is a lifesaving intervention for pregnant women and foetus [2].

ANC provides the opportunity to detect and treat complications of pregnancy and to deliver preventive health services such as immunisation against tetanus, prophylactic treatment of malaria and worms, and HIV testing and counselling leading to Prevention of Mother to Child HIV Transmission (PMTCT) [2–4]. An extensive body of evidence has shown that women who start ANC attendance early and continue regularly, are likely to get birth through the hand of a skilled birth attendant compared to those who initiate ANC late and attend few visits [5–8]. Beside the role of detecting risks factors, ANC exposes women to health education on danger signs and birth preparedness and encourages them to deliver with a skilled attendant or in a health facility [9]. Knowledge attained by pregnant women who attend ANC is not only used during the pregnant period but also after giving birth [9].

The World Health Organization (WHO) and the Tanzanian Ministry of Health’s ANC guidelines recommend at least four ANC visits for uncomplicated pregnancies with the first visit starting before 12 weeks of gestation [3, 10]. However, studies have indicated that the vast majority of women in sub-Saharan Africa start antenatal care considerably late [3, 11–14]. A comparative analysis of the use of maternal health services in sub-Saharan Africa showed that adolescent mothers initiated ANC attendance even later than adult mothers [11].

The findings of the recent Tanzania Demographic and Health Survey showed that though over 90% of pregnant women attend ANC at least once, only 51% make four or more visits during their entire pregnancy, and only 24% of women made their first ANC attendance before the fourth month of pregnancy [15]. In Dar es Salaam the figure is even lower than the national coverage. Only 14% of pregnant women started ANC clinics before the third month of pregnancy in Dar es Salaam [15].

Factors influencing utilization of antenatal care in general have been widely studied. It has been shown that mothers who are educated, and those whose husbands are educated are more likely to utilize antenatal care services [16–18]. Availability, affordability and easy accessibility of ANC services increase its utilization [17, 18]. Studies have reported that pregnant women with low socio-economic status are less likely to use antenatal care services than those with higher socio-economic status [19, 20].Cultural beliefs and ideas about pregnancy also have been reported to influence ANC use and may lead to mothers attending ANC late or attend a few ANC visits [21].Other studies have reported that younger age and unplanned pregnancy, and lack of knowledge regarding the importance of early ANC are the causes for delayed initiation of ANC [22–24].

While factors for the utilization of antenatal care in general have been widely studied, there is paucity of studies on the factors affecting timing of the first ANC attendance. Most previous studies on late ANC attendance have mainly relied on the use of questionnaires [21]. Although these studies provide insights into factors for late attendance of ANC, context-specific studies on individual, community and health system factors that lead to late initiation of ANC are needed. Only few studies have employed qualitative approaches to explore factors that influence ANC attendance in sub-Saharan Africa [22, 25–28]. These studies highlight the importance of understanding context-specific factors for ANC attendance. Using a qualitative approach, this study aimed to explore context-specific factors that lead to delay in seeking ANC services among pregnant women in Ilala Municipal in Dar es Salaam region, Tanzania.

## Methods

The study was conducted in Reproductive and Child Health Care clinics in three different health facilities in Ilala Municipal in Dar es Salaam region. According to the National Population Census of 2012, Ilala Municipal has1,220,611 inhabitants. It is the largest Municipality in Dar es Salaam Region. The population is comprised of different tribes from almost all regions of Tanzania. The study was done at Chanika, Kinyerezi and Arafa public health facilities. These facilities were randomly selected.

An exploratory qualitative study was conducted from April to June 2017. The use of exploratory design was based on its ability to offer an opportunity to explore people’s experiences in their own settings. The study was conducted by the first author (SM) as part of his Master of Public Health (MPH) programme at the University of Dar es Salaam. Interviews were conducted in Kiswahili language by the first author (SM). A purposive sampling technique was used to recruit respondents. Interviews were conducted with health care providers and with women who were attending antenatal care and women who had delivered in the last 12 months present in the clinic on the day of the visit. Interview guides were developed to assist semi-structured interviews with respondents. Interviews were audio-recorded with the permission of the respondents. The saturation point was reached after conducting in-depth interviews with 20 women attending ANC and PNC clinics and five interviews with health providers.

### Data Analysis

Thematic analysis approach was used to analyse the data. First, recordings of the interviews were transcribed verbatim by SM and checked for accuracy by a senior researcher SOM. Transcription and data analysis were carried out from July – September 2017, immediately after the field work. Interviews were translated from Kiswahili to English by a professional language expert. Both authors read the transcribed interviews in order to familiarise themselves with the data. Data were coded manually by SM in accordance with the identified themes. Additional codes which emerged during the coding process were added along the way. The second author (SOM) who is more experienced in qualitative research checked for consistency during the coding process. Data were summarised and synthesised, retaining key terms, phrases and expressions of the respondents. Finally, interpretation of the themes was realized by reading the transcripts several times.

## Results

Median age of the women was 26.4 years and the age range was 19 to 37 years. The majority of pregnant women were not married 8 (40.0%). Nine women (45.0%) of the women were in their first pregnancy, 15 women (75.0%) had a gestational age below 19 weeks and five (25%) were between 20 and 32 weeks. Thirteen women (65.0%) women had primary education, 5 (25.0%) secondary education, and only one (5.0%) had above secondary education. Table 1 summarizes the socio-demographic characteristics of the women.

**Table 1:** Socio-demographic characteristics of pregnant women

Variables	Number of women (*n* = 20)	Percent
Age group (years)		
16–24	10	50.0
25–34	7	35.0
35–44	3	15.0
Marital Status		
Married	7	35.0
Not married	8	40.0
Co habiting	5	25.0
Gestation age (Weeks)		
Up to-19	15	75.0
20–32	5	25.0
33 and above	0	0.0
Education Level		
No formal education	0	0.0
School drop outs	1	5.0
Primary education	13	65.0
Secondary education	5	25.0
Above secondary	1	5.0

Median age of healthcare workers was 38.6, ranging from 26 to 51 years. The majority of health care workers were nurses with four being female and one male. Table 2 shows the summary of the socio- demographic characteristics of the health care workers.

**Table 2:** Socio-demographic characteristics of health care workers

Variables	Number of Health care workers (*n* = 5)	Percent
Age in years		
24–34	2	40.0
35–44	1	20.0
45–54	2	40.0
Sex		
Male	1	20.0
Female	4	80.0
Title		
Nurse assistant	3	60.0
Nurse midwife	1	20.0
Registered nurse	2	40.0
Medical doctor	0	0.0

### Perceptions of women on ANC attendance

The majority of respondents expressed the importance of starting antenatal care as soon as pregnancy is confirmed. Respondents believed that early antenatal care attendance gives pregnant women an opportunity to be tested and advised to improve health and prevent harm to both mother and baby. Worries of uncertainty and pregnancy complications were reasons to start seeking antenatal care services immediately after confirming pregnancy. One respondent put it this way:

*“I have always had problems with my pregnancy. I got sick during my last two pregnancies…I am always worried that anything bad could happen. This is the third time I am starting my clinic at 3 to 4 months of pregnancy”*(IDI with a pregnant woman).

In addition, respondents said, HIV testing is one of the very preliminary tests done during the first antenatal care visit. Thus, early attendance enables one to understand her HIV status. For HIV positive individuals it helps the mother to start anti-retroviral therapy as part of prevention of mother to child transmission. However, a few respondents were not aware of the importance of early initiation of the ANC. One respondent illustrated this way:

*“It is not bad to start antenatal care early. But for me, if you don’t have any problems with your pregnancy, there is no need to start early. Let others with problems go and get help”* (IDI with a pregnant woman).

A number of individual factors resulted in late ANC attendance. The key themes which emerged were as follows:

#### Lack of awareness and misconceptions of pregnant women

Most pregnant mothers especially during the first pregnancy delayed ANC attendance because they were not aware when to start ANC clinics and the number of visits recommended as exemplified by one responded:

*“I started my first clinic at fifth month of pregnancy. I thought if you start early you will have to attend more visits. I didn’t know it is the same four visits. I just learned this after starting the antenatal care clinic*” (IDI with a woman with a child).

A few respondents reported that lack of familiarity with the signs of pregnancy encouraged hesitation to disclose pregnancy. As a result, women were more likely to recognise pregnancy at a later stage and thus unintentionally delayed ANC attendance. Some respondents pointed this way:

*“I was using injections for family planning. Therefore, for a very long time I had not seen any menstrual flow. So even after conceiving I didn’t notice. I had no other signs until when my stomach started budging”* (IDI with a pregnant woman)*.*

Furthermore, health care workers highlighted misperceptions and lack of information, especially among low income families, regarding costs for antenatal care. It was reported that some families thought that ANC services are charged and thus they could not afford the costs of registration, testing for HIV and other diseases, medicine, and consultation. In reality these services are not charged for antenatal care.

While antenatal care services in Tanzania are provided free, women reported some indirect costs which resulted into delayed ANC attendance. A few respondents reported that women needed some pocket money to spend during clinic days. This is particularly important because in most cases women spent almost the whole day when attended ANC clinics*.*

Women with experience of smooth pregnancy and delivery delayed ANC attendance as they felt that everything would be all right. One respondent illustrated this way:

*“My mother delivered all of us at home. I don’t see any problem starting antenatal care late as long as you’re doing fine”* (IDI with a pregnant woman)*.*

#### Distance to the health facility

Women from poor families living far from health facilities could not afford transport costs and choose to wait until delivery time. One respondent put it this way:

*“Some health facilities are very far. You need to have a bus or bodaboda (*motorbike) *fare to get there. Most poor families cannot afford this for all visits. When you are waiting to get money time also passes”*(IDI with a woman).

This finding was also confirmed by health care workers. Particularly transport made pregnant women delay starting ANC visits and even sometimes not attending completely. One respondent put it this way:

“*Distance from facilities is one of the problems. There are some who have to take bodaboda every visit. Most of them choose to delay or even skip clinic”* (IDI with a health worker).

#### Socio - cultural beliefs

All types of respondents reported that adolescents and unmarried younger women tend to hide their pregnancies to avoid potential problems like exclusion from school, stigmatization and gossip. As a result they delay ANC attendance. Some women feared to disclose their pregnancy early due to witchcraft.

*“I started antenatal clinic late because my mother- in- law told me to wait for a couple of months. She said if I disclose my pregnancy early, my child can disappear supernaturally”* (IDI with a pregnant woman)*.*

Given the social nature of pregnancy, women preferred to look smart while attending ANC visits. As a result, women who were not able to get new clothes on time were forced to delay ANC attendance. One respondent said this way:

*“You know for us women, when you want to go to the public and you don’t have good or new clothes, you feel shy and very uncomfortable. This makes some women delay antenatal care clinic”* (IDI with a pregnant woman)*.*

### Community barriers for late ANC attendance

There were some community factors that influenced the behaviour of pregnant women for early ANC attendance.

Respondents highlighted the importance of support from peers and family members. In some cases, support was good and prompted early ANC visits. In others, the influence of peers resulted women to delay ANC visit. One respondent illustrated this way:

*“Because it was my first time, I asked my friends. But they threatened me that if you start early you will get tired… I was scared and didn’t know what to do until I met someone with experience who was planning to go to clinic the next day. We then went together”* (IDI with a pregnant woman)*.*

Most community members were supportive of antenatal care, although there were a few members of the community who discouraged pregnant women to start ANC early. Some women were worried of witchcraft especially during the early months of pregnancy. One respondent said this way:

*“In my community, they totally discourage to start clinic when the pregnancy is below four months. They say that there are people with bad eyes who can destroy your pregnancy. I started antenatal care when my pregnancy was 6 months. My mother is very strict on this”.* (IDI with a pregnant woman).

### Health system barriers for late ANC attendance

Respondents highlighted a number of health system related factors which made women start ANC late.

Focused antenatal care guidelines require escort of a spouse during the first ANC visit. The aim of this is to enable health care workers provide health education to the couple on danger signs during pregnancy, individual birth preparation, and more important counselling and testing for HIV.In order to implement this policy, health facilities have made it mandatory for a woman to bring her spouse during the first ANC attendance. Pregnant women who are not able to come with their partner were required to get an introductory letter from the village/street leaders otherwise they may be denied antenatal care services. Respondents reported that pregnant women who had challenges to get male partner’s support for different reasons felt reluctant to start ANC attendance as they were worried that they would be denied antenatal care services.

*“I have my friend and we wanted to start clinic together. Unfortunately, she was dating someone’s husband and the man could not accept going with her to the clinic. So she had to wait before we figured out something”*(IDI with a pregnant woman).

Another respondent added this way:

*“I was sent back home two times because I did not come with my spouse. I had to convince someone to escort me. Then I was allowed to start clinic”* (IDI with a pregnant woman).

This finding was confirmed by health providers. While health providers were aware of the unintended consequences of this policy they had the opinion that the policy was good for the health of the pregnant mother and the new baby. One respondent put it this way:

*“We insist they come together so that we can educate, counsel and test them….We usually deny services pregnant mothers who come alone, especially when we believe that it is possible to bring her spouse. I think this contributes to the late ANC attendance”* (IDI with a health worker).

However, health providers reported dilemmas they were facing in deciding to deny services to pregnant women who did not come with partners. One respondent exemplified it this way:

*“It is very difficult to decide to send a client back home. But sometimes it is inevitable. You have to make sure they come together so that they get health education together and test for HIV”* (IDI with a health provider).

Respondents reported that pregnant women and their spouses who were not sure of their HIV status were afraid of starting ANC attendance. Requirement for HIV testing was the main factor for low male partners escort for antenatal care. One respondent explained this way:

“*Many people are worried to do HIV test. They are compelled to go late, especially when they start experiencing problems with pregnancy.”* (IDI with a woman).

Respondents highlighted the worries mothers have in receiving HIV test results especially when it is HIV positive and the difficulty to accept the situation and cope with life after the disclosure of the test results.

*“During my second pregnancy, I never attended a clinic. I gave birth at home, but it was not easy. That’s why this time I decided to come. The reason I didn’t come was that I was suspecting weird results. I suspected my husband had many other sexual partners”* (IDI with a pregnant woman)*.*

It was also reported that pregnant women who were aware of their HIV status avoided going early to antenatal care clinics because of fear of disclosure of their HIV status to the health providers and other people. Fear of stigma delayed pregnant women who were HIV-positive to start antenatal clinic. This finding resonances with the interviews with health care workers. One respondent put it this way:

“*Women who know their HIV status do not like to test with their spouse and share results. But our requirement is for them to come together. Most of them will delay ANC attendance and will make sure they come alone or never come for ANC services”* (IDI with a health worker).

Health facilities had critical shortage of health providers.

In almost all facilities, health care workers complained that they were overwhelmed by workload. One respondent said this way:

*“As you can see, today I am alone. My colleague has gone to the district office. We are only two. You can see the long queue outside”* (IDI with a health worker).

Shortage of health providers prompted health facilities to schedule specific time for antenatal care. In order to serve many women at a time, in most cases, health education prior to the counselling and testing was provided in groups. Shortage of health providers resulted into long waiting times. The vast majority of women could manage to stay long in the health facilities waiting for the services, men could not tolerate. In most cases, men were reported to be the principal breadwinners of the household. It was thus very challenging for men to sacrifice income generating activities for virtually a whole day.

Respondents complained about the behaviour of some health care workers to treat them with no respect by shouting, reprimanding and talking to them as non-adults even when the mothers were older than health care workers.

*“Sometimes, the nurse just shouts at you and you feel very bad because most of them are our children. When you recall this, you feel like not going to clinic”*(IDI with a woman with a child).

A few respondents mentioned the behaviour of some health care workers to laugh at pregnant teenagers coming to start antenatal care discouraging others as they go back and discuss with peers. This behaviour discourages early attendance.

## Discussion

The findings of the study revealed young and adolescent pregnant women also started ANC attendance late due to various factors. This finding is consistent with the literature. For example, a comparative study conducted in Kenya, Ghana and Malawi reported that adolescent and unmarried young women hid their pregnancies and delayed ANC to avoid the potential social implications of pregnancy, exclusion from school, expulsion from their home, partner abandonment, stigmatization, and gossip [27]. Similarly, ethnographic studies from Mozambique and Southern Tanzania illustrated that women at an early stage of pregnancy delayed ANC initiation in order to protect the unborn from witchcraft and sorcery attacks of jealous neighbours [25, 29]. In Zimbabwe pregnant women feared to attend ANC during the first trimester due to the local belief that the early period of pregnancy was most vulnerable to witchcraft [26].

This study indicated that community members still consider pregnancy as a mysterious event that should not be announced early. These findings underline the need of a continued focus on sensitization campaigns on the importance of early initiation of ANC clinics. The focus should be on socio-cultural barriers to early ANC attendance. Religious leaders should be sensitized and capacitated with key messages that can be used to address socio-cultural beliefs and misconceptions [3–32].

Geographic unaccessibility contributed to late initiation of the ANC attendance. This finding has also been reported in other studies [9, 21, 33, 34]. Nsibu and colleagues demonstrated statistical significant associations between place of residence and the first ANC visit in the first trimester [33]. We recommend that district health managers should ensure that ANC services are brought closer to where people live. This could be achieved through mobile clinics, particularly in rural and hard to reach areas. This would help many women who are facing economic difficulties with access to ANC.

The finding of this study revealed that while spouse’s escort and HIV testing policy was intended to encourage men to accompany their partners during ANC, the policy compelled women to delay ANC attendance and sometimes completely skipping ANC. While this policy is important for promoting PMTCT and other maternal and child health outcomes, the implementation of the policy should take into consideration the culturally deep-rooted gender roles in the community [35]. In a society where pregnancy and childbirth matters had for a long time been considered as women’s affairs, it should not be an obligation for a woman to come with her partner. When women come alone they should be able to get services without hesitation or bad language.

Interpretation of the spouse’s policy should not solely be left to the health providers who may only seek to achieve health related output targets such as increasing the number of couples who take HIV test while ignoring social consequences of this policy. The district and regional health officials should provide correct interpretation of this policy and provide intensive sensitization to the policy implementers on the importance of taking into consideration the ingrained gender roles in maternal and child health. In addition, community members, men in particular, should be sensitized on the importance of ANC timing and HIV testing during pregnancy.

Instead of denying women services, health providers should use verbal and official invitation to request men to accompany their partners during the next ANC visit. For example, in Malawi while mandatory HIV testing was part of routine antenatal care, a woman who visited the clinic for the first ANC visit, was requested to come with her husband during the next visit [32]. Invitation letters have been widely used to promote male participation in PMTCT in various settings [36–40]. Invitations should highlight the importance of ANC visits for men.

Our findings revealed that fear for HIV testing for both pregnant women and men resulted into late ANC attendance. Earlier studies have reported that men and women fear to receive HIV positive results due to the HIV-associated stigma and disclosure [41–45].While studies have reported that with the availability of life-prolonging antiretroviral therapy, people tend to accept HIV status and that HIV is now becoming accepted in the society [41, 42], perceived stigma seems to continue influencing individuals’ decisions to disclose their HIV status [41, 45–47]. This finding underscores the need of further sensitization of community members with regard to the importance of knowing their HIV status. Furthermore, efforts done by the government, non-governmental organizations and other stakeholders to address stigma related to HIV disclosure should be continued.

Equally important, health worker training and system strengthening have the potential to increase the quality of ANC services. In order to fully implement ANC components such as counselling and integrated testing and treatment for PMTCT of HIV, a sufficient number of adequately trained health care workers is necessary. By addressing these supply side factors, the perception of quality of ANC care and the rate of male involvement in pregnancy and childbirth may improve. For example, creating “male-friendly” waiting areas, extended hours of providing ANC services, and reducing waiting time in health facilities have been reported to increase male participation in maternal and child health services [34, 48–50].

### Strengths and limitations of the study

Key strength of this study is that data were collected from multiple respondents including health providers, pregnant women, and women who had delivered recently. This provided a good opportunity to validate the findings from different categories of respondents. Further, a qualitative approach provided opportunity to capture perceptions and experiences of the respondents in their natural social settings. Notwithstanding these strengths, limitations of the study are that interviews were only conducted with women. It is possible that interviews with men would provide additional or different information on their perspectives regarding factors that lead to delay in seeking antenatal care. In addition, this study was conducted in one district and the findings may not adequately reflect experiences of other districts in Tanzania. While more studies covering different geographical settings would be helpful for evidence based policy-making, this study provides an in-depth understanding of various factors which influence the timing of the first ANC attendance.

## Conclusion

This study aimed to explore factors for late initiation of ANC attendance in Dar es Salaam, Tanzania. This study concludes that individual, community and health system related factors played a key role affecting timing of ANC attendance. Community members should be sensitized about the importance of early ANC attendance and HIV testing during routine ANC attendance. While spouse’s escort policy is important for promoting PMTCT and other maternal and child health outcomes, interpretation of the policy should not solely be left to health care providers who may only seek to increase the number of couples who take HIV tests while ignoring social consequences of this policy. Further, the government should expand provision of ANC services near to its population to reduce geographical and indirect costs associated with ANC attendance. This could be achieved through mobile clinics, particularly in rural and hard to reach areas.

## Data Availability

The datasets generated and analysed during this study are not publicly available since participants did not give consent for the public sharing of their information. However, summaries of the information are available from the corresponding author upon request. The interview guides for all study participants are also available upon request.

## Abbreviations

AIDS: Acquired Immune Deficiency Syndrome
ANC: Antenatal Care
HIV: Human Immunodeficiency Virus
IDI: In-depth interview
MCH: Maternal and Child Health
PMTCT: Prevention of Mother to Child Transmission
WHO: World Health Organization
